# Emerging Technology in Refractive Cataract Surgery

**DOI:** 10.1155/2016/7309283

**Published:** 2016-06-28

**Authors:** João Saraiva, Kristin Neatrour, George O. Waring IV

**Affiliations:** ^1^Storm Eye Institute, Medical University of South Carolina, Charleston, SC 29425, USA; ^2^College of Engineering and Science, Clemson University, Clemson, SC 29634, USA

## Abstract

Technology in cataract surgery is constantly evolving to meet the goals of both surgeons and patients. Recent major advances in refractive cataract surgery include innovations in preoperative and intraoperative diagnostics, femtosecond laser-assisted cataract surgery (FLACS), and a new generation of intraocular lenses (IOLs). This paper presents the latest technologies in each of these major categories and discusses how these contributions serve to improve cataract surgery outcomes in a safe, effective, and predictable manner.

## 1. Introduction

With cataract surgery regarded as the most widely performed surgical procedure, a demand exists for continued innovation and technology. The latest advances evolved through application of well-defined principles to current surgical goals and patient expectations. For example, femtosecond laser technology emerged after fifty years of employing laser technology in ophthalmology [1]. Theodor Scheimpflug described the principle of scheimpflug images in 1904 [2], but he was actually an Austrian army captain who spent his life's work dedicated to designing methods and tools to create maps depicting aerial photography [3]. Application of these principles to ophthalmology in the last few years has advanced our understanding of corneal biomechanics [4]. The latest highlights in technology include advances in preoperative and intraoperative diagnostics, femtosecond laser-assisted cataract surgery (FLACS), and a new generation of intraocular lenses (IOLs).

## 2. Preoperative and Intraoperative Diagnostics

More than ever, patients have the desire to reduce their dependence on spectacles after cataract surgery. Physicians now have access to advanced diagnostics that can better quantify conditions such as dry eye, light scatter, and posterior corneal astigmatism. These technologies can enhance refractive measurements and appropriate IOL selection.

**Figure 1:**
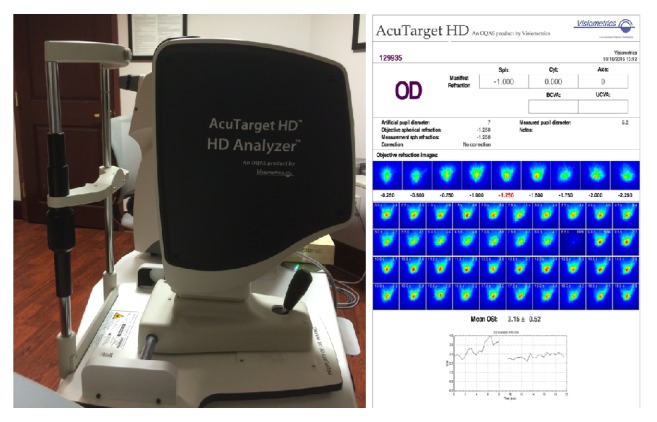
The AcuTarget HD and representation of objective scatter index and tear film analysis.

McDonald recently reported that the postoperative prevalence of dry eye related symptoms is approximately 88% [5]. Analysis and optimization of dry eye preoperatively and postoperatively has a beneficial impact on visual outcomes after cataract surgery [6]. Therefore, increased interest among ophthalmologists to utilize objective measurements to assess the ocular surface exists. The Acutarget HD (Visiometrics SL, Spain) assesses the objective scatter index, which can objectively evaluate dry eye disease severity using the degradation of image quality over time (Figure 1) [7]. The Keratograph (Oculus, Germany) noninvasively measures tear break up time, tear meniscus height, and meibography, providing a functional and qualitative analysis of the corneal surface and tear film [8, 9]. The TearLab Osmolarity System (TearLab Corporation, San Diego, California) uses a small tear sample to measure tear osmolarity using a microelectrode. Compared to other commonly used diagnostic tests for dry eye disease, test results were better at predicting dry eye severity [10]. The Lipiflow (TearScience, Morrisville, North Carolina) combines heat and eyelid pressure to treat dry eye disease due to meibomian gland dysfunction. Recent studies showed consistent improvement in meibomian gland function up to 12 months after the treatment [11].

Evaluation of optical quality also aids in decision making between corneal or lens-based procedures. The C-Quant (Oculus, Germany, OptikGenrate GmbH) assesses straylight subjectively by utilizing a compensation comparison method [12]. The Acutarget HD (Visiometrics SL, Spain) uses a double pass system to measure point spread function (PSF), modulation transfer function (MTF), Strehl ratio, and intraocular scattering of the light. These data allow clinicians to evaluate the quality of a patient's optical system objectively [13]. Another objective functional diagnostic is the Salzburg Reading Desk (SRD Vision, Vienna, Austria), which allows measurement of the variable read print sizes and distances with differences in contrast sensitivity and luminance [14].

Both anterior and posterior corneal astigmatism should be taken into account in IOL planning, particularly in patients desiring astigmatic correction. Inaccuracies arise when posterior corneal astigmatism is measured based on the assumption of a fixed-ratio relationship with the anterior curvature [15]. The Cassini Corneal Shape Analyzer (i-Optics BV, The Hague, The Netherlands) is a new topographer that uses LED ray tracing technology with 700 diode lights to measure anterior and posterior corneal astigmatism. These advances in cylinder and axis measurement precision can be useful for preoperative planning of toric IOL implants and in postrefractive surgery patients [15–17].

**Figure 2:**
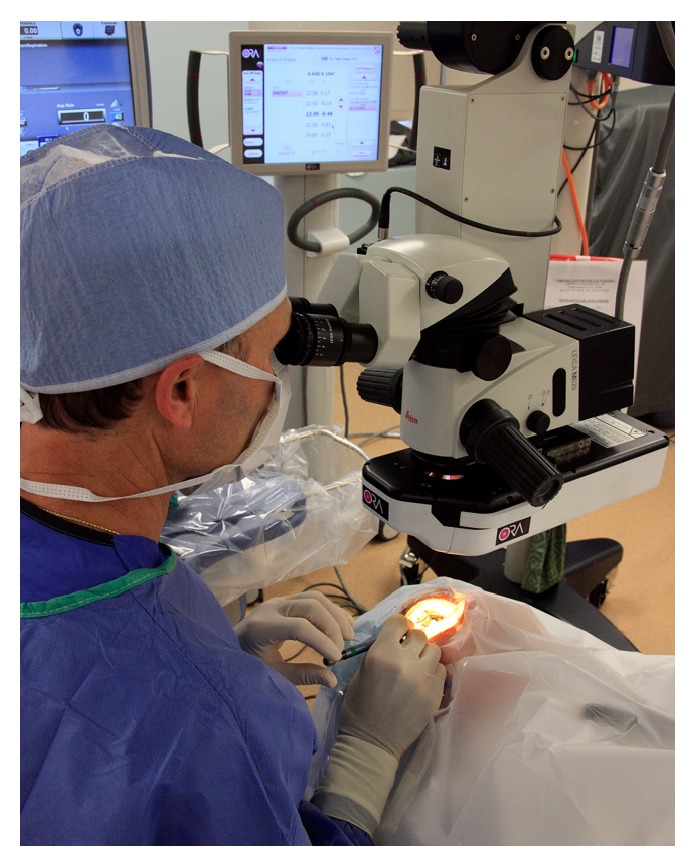
The ORA device attached to the operating microscope.

Patients with a history of corneal refractive surgery expect reduced dependence on spectacles after cataract surgery. The use of intraoperative aberrometry can adjunctively guide the future of IOL power selection [18]. The Optiwave Refractive Analysis (ORA, Alcon, Fort Worth, TX) uses wavefront interferometry to produce a fringe pattern, and distortions in this pattern are translated into refractive values and aphakic and pseudophakic readings (Figure 2) [19]. Hatch et al. studied mean postoperative residual refractive astigmatism in patients receiving toric IOLs with power selection aided by intraoperative aberrometry. Surgeons altered cylindrical power 24% of the time and spherical power 35% of the time. Patients were 2.4 times more likely to have less than 0.50 D of residual refractive astigmatism when intraoperative aberrometry was used [20]. In contrast, Huelle et al. published a study where aphakic spherical equivalent- (SE-) based IOL formulas were generated from repeated intraoperative wavefront aphakic measurements of SE. The agreement of repeated aphakic SE readings ranged from −0.69 diopters to +0.66 diopters. The authors concluded that measurement precision is limiting reliability of intraoperative aberrometry and application to routine cataract surgery [21]. However, it may be useful in guiding limbal relaxing incision enhancements and has resulted in the need for fewer subsequent laser enhancements [22]. This technology is particularly useful in postrefractive patients and those with astigmatism uncertainty or other corneal pathology. However, barriers exist, such as financial, temporal, and workflow considerations. Other intraoperative inconsistencies include cyclotorsion, variable anterior chamber depth and intraocular pressure, variability in wound hydration, and use of viscoelastic device versus balanced salt solution [18]. Although limitations may exist in quality and measurement precision, the future of this technology is promising.

The use of the electroretinogram (ERG) has been well described and may have a novel application for refractive cataract surgery. Dr. Richard Mackool described the use of flash ERG testing with office-based electroretinography (Diopsys, Pine Brook, New Jersey) in preoperative cataract evaluation. It can provide an objective evaluation of macular function and could be useful in influencing lens selection for patients with conditions such as epiretinal membrane, diabetic retinopathy, and age-related macular degeneration. More studies evaluating ERGs in preoperative cataract assessment need to be done to further assess its value and implications [23].

## 3. Femtosecond Laser-Assisted Cataract Surgery

**Figure 3:**
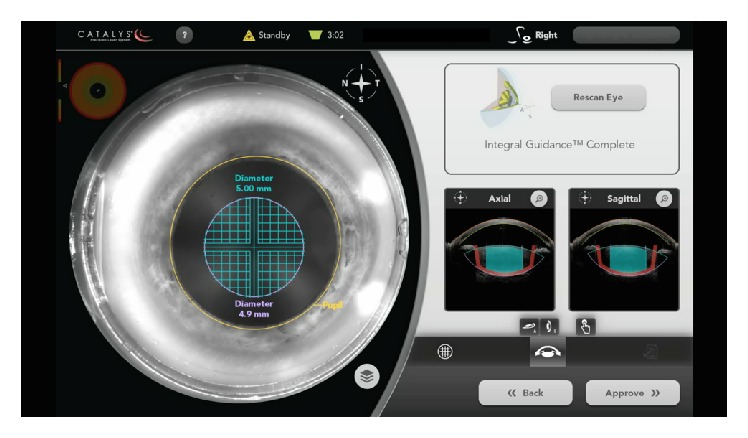
The Catalys femtosecond laser interface.

Femtosecond laser-assisted cataract surgery (FLACS) has realized increasing popularity. Noninferiority has been established relative to manual cataract surgery, and some reports have suggested superiority relative to manual methods [24]. Potential advantages include customized corneal incisions and capsulotomy position, precision in shape and size of capsulotomy, custom lens fragmentation patterns, endothelial cell loss reduction, and better refractive stability and predictability [24]. After the Food and Drug Administration (FDA) approval of laser-assisted capsulotomy and lens fragmentation in 2010, five platforms have been released: LenSx by Alcon (Fort Worth, TX); the LensAR by LensAR (Orlando, FL); the Catalys by Abbott/Optimedica (North Chicago, IL); the Victus by Bausch and Lomb (Rochester, NY); and the LDV Z8 by Ziemer (Port, Switzerland) (Figure 3) [25].

The docking process using the femtosecond laser-eye interface uses a suction ring to stabilize the eye, thereby allowing imaging and laser delivery through a clear optical pathway. Considerations for docking include complete coupling, patient comfort, intraocular pressure elevation, and minimal distortion of anatomy to avoid disruption of the beam path. In a study using Alcon's LenSx platform to compare curved direct contact and modified soft interfaces (SoftFit*™* by Alcon), Mayer et al. showed that redocking was unnecessary when a modified soft interface was used, even though some cases resulted in incomplete incisions requiring manual opening [26]. Schultz et al. found significantly fewer intraocular pressure elevations after docking using a liquid interface (Liquid Optics interface, Catalys Precision Laser System) in comparison to flat and curved interfaces [27]. While docking is a necessary step with femtosecond laser technology, laser incisions are optional in FLACS. Incisions with predictable morphology and sealant features can decrease incision-related adverse effects [28]. Mastropasqua et al. comparatively analyzed femtosecond laser incisions and manual incisions and cited better tunnel morphology with FLACS incisions [28].

FLACS theoretically decreases endothelial cell loss relative to manual techniques by reducing the use of ultrasound energy. However, Krarup et al. compared endothelial cell loss rates between phacoemulsification and FLACS and showed there were no differences between both modalities [29]. Abell et al. published similar findings but did cite a difference in favor of FLACS that was limited to the early postoperative period. They also showed that laser corneal incisions themselves may influence endothelial cells, as there may be a disturbance in the postoperative inflammatory response after laser application [30–32]. New surgical techniques, in combination with more advanced lens fragmentation patterns, will allow the lens to be extracted through an aspiration mechanism that may reduce endothelial cell loss.

**Figure 4:**
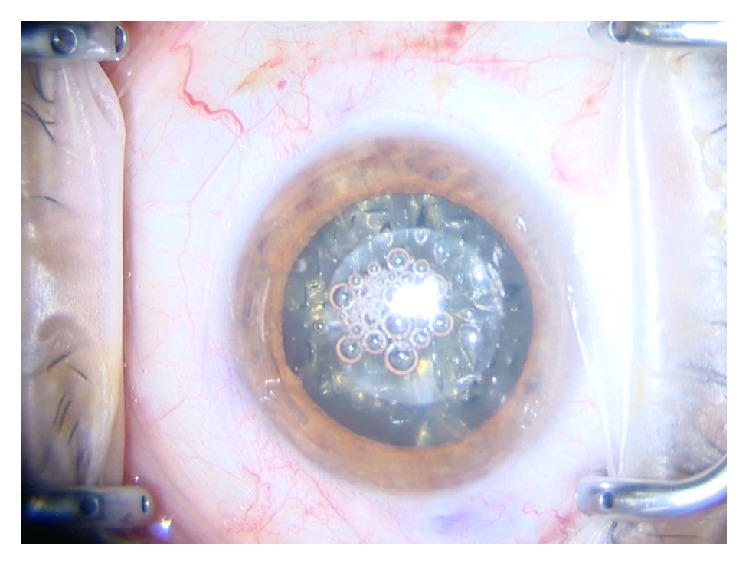
Surgeon view of eye after femtosecond laser capsulotomy lens softening and limbal relaxing incision with the Catalys laser.

The size, shape, and position of a capsulotomy should theoretically lead to a more predictable lens position by enhancing uniform capsule-optic overlap, thereby reducing the incidence of lens tilt and leading to an overall better effective lens position and visual outcome (Figure 4). Recently, Toto and colleagues found no difference in prediction error when comparing traditional phacoemulsification with FLACS but did find higher refractive stability and IOL centration with FLACS [33]. This similarity in prediction error may be a consequence of unexplored potential with IOL calculations and algorithms. Dr. Ma approached the prediction of true lens position using an algorithm based on OCT anterior segment 3-D reconstruction [34]. This prediction model could have great potential once there is consistent alliance of OCT measurements with FLACS to provide more precise outcomes. This is particularly relevant with premium IOLs, as there is a lower tolerance threshold for minor unanticipated miscalculation and decentration. Okulix (Tedics Peric & Joher GbR, Dortmund, Germany) is an innovative software program that calculates IOL power using ray tracing combined with corneal topography. Saiki et al. evaluated its accuracy in post-LASIK eyes in comparison with Camellin-Calossi, Shamas-PL, Haigis-L formulas and double-K SRK-T method. They reported that this technology provides sufficient predictability outcomes in postrefractive myopic LASIK, even though a small hyperopic shift tendency was noted in the study [35]. The Galilei G6 Lens professional (Ziemer, Port, Switzerland) is an optical biometer that integrates Placido rings with a dual rotating Scheimpflug camera as well as an optical coherence tomography based A-scan in a single device. Shin et al. compared its accuracy with the Lenstar LS 900 (Haag-Streit, Koeniz, Switzerland), for intraocular lens (IOL) power calculation. They noted that axial length, lens thickness (LT), and white-to-white (WTW) values were statistically different. Thus, even though high repeatability was present, and the IOL powers were not statistically different between the two devices, the values provided by the Galilei G6 were not interchangeable with the Lenstar in the clinical setting [36].

## 4. Intraocular Lenses 

The goal of appropriate IOL selection is to provide the best visual outcome that meets a patient's individualized goals and expectations. Variability in materials, optical properties, and designs are important factors to consider in the patient-specific selection of an IOL. Advancements in IOL technology aim to improve visual functionality by creating customized IOLs or modifying optical power postoperatively.

The concept of adjustable IOLs involves the correction of residual refractive error postoperatively or customization after lens implantation. This new paradigm in IOL manufacturing may be subdivided into two major categories: a modular multicomponent category requiring a separate intraocular procedure and another category where the optic is adjusted postoperatively with a secondary device. The first category includes multicomponent IOLs (Clarvista Harmoni modular IOL system, Clarvista Medical, Aliso Viejo, CA; and Omega Lens, Omega Ophthalmics, Lexington, KY); Infinite Vision IOL (Infinite Vision Optics, France); and mechanically adjustable IOLs (Acritec AR-1 PC/IOL, Acri.Tec, Hennigsdorf, Germany). These are currently being studied in vivo with promising preliminary results. The second category includes magnetically adjustable IOLs (University of Missouri-Rolla, Rolla, and Eggleston Adjustable Lens, St. Louis, MO), light adjustable IOLs (Calhoun Vision, Pasadena, CA), and the Perfect Lens (Perfect Lens, LLC, Irvine, CA). The latter is a novel platform, which can be adjusted with the femtosecond laser based on the concept of refractive index shaping. The light adjustable IOL is currently in FDA clinical trials. Its mechanism involves the use of infrared light to polymerize photosensitive silicone macromers, which results in changes in lens morphology and optical properties [37].

A new generation of optics with extended depth of focus, multifocal rotational symmetry and asymmetry, and accommodating capabilities offers promising strategies to advance functional vision in refractive cataract surgery. The Tecnis Symfony (AMO) is an extended depth of focus IOL that works by correcting chromatic aberration using diffractive optics and reduces glare and halo symptoms classically associated with conventional multifocal IOLs [38]. Multifocal lenses with bifocal and trifocal designs include the rotationally asymmetric Lentis Mplus (Topcon Europe Medical BV, The Netherlands) and the rotationally symmetric FineVision (PhysIOL, Liege, Belgium) and AT LISA (Zeiss, Oberkochen, Germany). Mojzis et al. found that trifocal lenses provide satisfactory intermediate vision without compromising near and far distance visual acuity [39].

Accommodating IOLs aim to simulate the natural mechanism of accommodation. The FluidVision IOL (PowerVision, Belmont, CA) has silicone oil inside the lens that moves in response to ciliary contraction forces. An electroactive IOL with a liquid crystal that is sensitive to electric current is also in development (Sapphire AutoFocal IOL, Elenza, Roanoke, VA). Dual accommodating IOLs designed for sulcus placement include the DynaCurve IOL (NuLens, Israel) and the Lumina IOL (Akkolens, The Netherlands) [37]. The injectable polymer SmartIOL (Medennium, Irvine, CA), which is a thermodynamic, pliable capsule-filling IOL, is the only bag filling technology in development [40].

The future of refractive cataract surgery is exciting; in time, these new technologies may be the standard of care. With refinements of the latest technology, FLACS and other parallel advances will provide surgeons with the potential to perform an even safer, predictable, and effective surgery.
